# Sociodemographic and clinical factors influencing typhoid fever prevalence and multidrug resistance in Niger State, Nigeria

**DOI:** 10.1371/journal.pone.0327740

**Published:** 2025-07-08

**Authors:** Dickson A. Musa, Hussaini Majiya, Umar A., Shedrack E. Akor, Salamatu Mambula-Machunga, Harun K. Aremu, Enyo Sule, Samuel E. Abah, Vincent T. Balogu, Andrew C. Iloh, Stella I. Smith

**Affiliations:** 1 Trans-Saharan Disease Research Centre, Ibrahim Badamasi Babangida University, Lapai, Niger State, Nigeria; 2 Department of Biochemistry, Ibrahim Badamasi Babangida University, Lapai, Niger State, Nigeria; 3 Department of Microbiology, Ibrahim Badamasi Babangida University, Lapai, Niger State, Nigeria; 4 Prince Abubakar Audu University, Anyigba, Kogi State, Nigeria; 5 Department of Microbiology, University of Abuja, Abuja, Nigeria; 6 Department of Biological Sciences, Mississippi State University, Starkville, United States of America; 7 Nigerian Institute of Medical Research, Lagos, Nigeria; 8 Department of Health, Science and Education, Coventry University Group, London Campus, London, United Kingdom; 9 Advanced Biotechnology, Sheda Science and Technology Complex, Abuja, Nigeria; Federal University Oye-Ekiti, NIGERIA

## Abstract

**Background:**

This study investigated the prevalence of typhoid fever and the factors contributing to multidrug resistance in Niger State, Nigeria, a region affected by limited sanitation and healthcare access.

**Methodology:**

A cross-sectional study design was employed across communities in Niger State, Nigeria, where participants in different communities of the state aged 18 years and above were enrolled. *Salmonella enterica* serotype Typhi isolation from stool and determination and interpretation of multi-drug resistance were performed according to the Clinical Laboratory Standards Institute (CLSI) methods. We examined the associations between sociodemographic characteristics, clinical factors, and multidrug resistant (MDR) in *Salmonella enterica* serotype Typhi.

**Principal findings/conclusions:**

Among the 624 participants, typhoid fever prevalence was recorded at 36.5%, with young adults (ages 18–27) and semi-urban residents demonstrating higher infection rates. Antibiotic sensitivity testing revealed 98% resistance to amoxicillin/clavulanic acid, whereas gentamicin and levofloxacin exhibited high efficacy. Our findings underscore the urgent need for health education, improved antibiotic regulation, and Water, Sanitation, and Hygiene (WASH) interventions to control typhoid fever and mitigate MDR challenges.

## Introduction

Typhoid fever remains a significant public health issue in low- and middle-income countries, particularly in sub-Saharan Africa, with an estimated 7.2 million cases annually [1]. A 2024 study estimated 144.6 cases per 100,000 person-years in SSA [2] and most importantly, children under 15 years are disproportionately affected, with incidence peaking in those aged 2–4 years [3]. In Nigeria, a 2019 systematic review found that the prevalence of typhoid fever ranged from 2% to 50%, depending on the region and diagnostic method [4].

The disease is caused by *the Salmonella enterica* serovar Typhi and is typically transmitted via contaminated food and water, exacerbated by inadequate sanitation and hygiene [5–7]. In Niger State, Nigeria, limited access to potable water, suboptimal waste disposal practices, and indiscriminate antibiotic usage have collectively contributed to consistent disease prevalence and an increase in multidrug-resistant strains of *Salmonella* Typhi [8,9]. Multidrug-resistant bacterial infections, particularly in *Salmonella*, due to first-line antibiotics pose significant challenges to global health. Multidrug resistant (MDR) strains are associated with more severe outcomes, including increased bloodstream infections, hospitalizations, and mortality, than susceptible strains [10].

Understanding the interplay between sociodemographic and clinical factors in typhoid prevalence and antibiotic resistance patterns is crucial for formulating targeted interventions. Although studies have elucidated the influence of age, sex, residence, and socioeconomic factors on typhoid transmission [11,12], limited research has specifically analyzed these parameters in relation to MDR patterns in Niger State. This study addresses these gaps by examining both the prevalence and resistance dynamics of typhoid fever within a comprehensive sociodemographic framework, aiming to inform public health policies tailored to the unique epidemiological landscape of Niger State. This study aimed to assess the prevalence of typhoid fever and the sociodemographic and clinical factors associated with MDR in Niger State, Nigeria, to provide insights for interventions that can mitigate transmission and resistance.

## Materials and methods

### Ethics statement

Ethical approval was obtained from the Ministry of Health, Niger State, Nigeria (ERC PAN/2023/07/27), and The Research Ethics Committee of Ibrahim Badamasi Babangida University, Lapai, Nigeria (Reference No.: IBBUL/01/2022). Participation in the study was voluntary, and all participants were requested to provide written informed consent before they were included in the study.

### Study area

**Fig 1:**
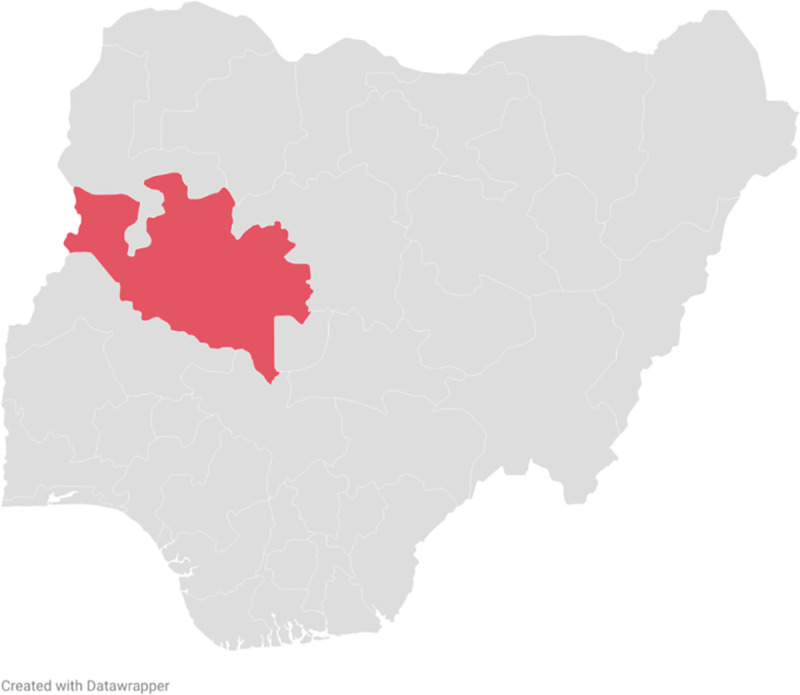
Map of Niger State, Nigeria.

Niger State, one of the 36 States of Nigeria, situated in central Nigeria between latitudes 8° and 11° north and longitudes 3° and 7° east, is the largest state in the country, covering 76,363 square kilometers (Fig 1). It borders the Federal Capital Territory (FCT) to the southeast and states, including Kwara, Kogi, Kaduna, Kebbi, and Zamfara. The Niger State also shares an international border with the Republic of Benin to the west. Niger State has an estimated population of approximately 6.8 million in 2022 based on the 2006 census [13]. Niger State has 25 local government areas with Minna as the capital city. The topography of the state includes diverse landscapes, such as the Niger River, plains, hills, and savannah grasslands, supporting various agricultural activities. The economy is primarily agrarian, with most of the population engaged in subsistence farming. The state is home to diverse ethnic groups, including Nupe, Gwari (Gbagyi), and Hausa. Niger State, characterized by diverse communities with varying socioeconomic statuses and living conditions, provides a comprehensive context for examining the prevalence of typhoid fever and multidrug resistance.

### Study design and setting

A cross-sectional study design and a random sampling method were employed to assess the sociodemographic and clinical factors influencing typhoid fever prevalence and multidrug resistance. The investigation was conducted across various communities within Niger State, Nigeria. Ethical clearance was obtained from the Niger State Ministry of Health and the participants provided informed consent. The cross-sectional design facilitated the collection of data at a single point in time, enabling a snapshot of the health status and risk factors of the population.

### Population and sample collection

**Fig 2:**
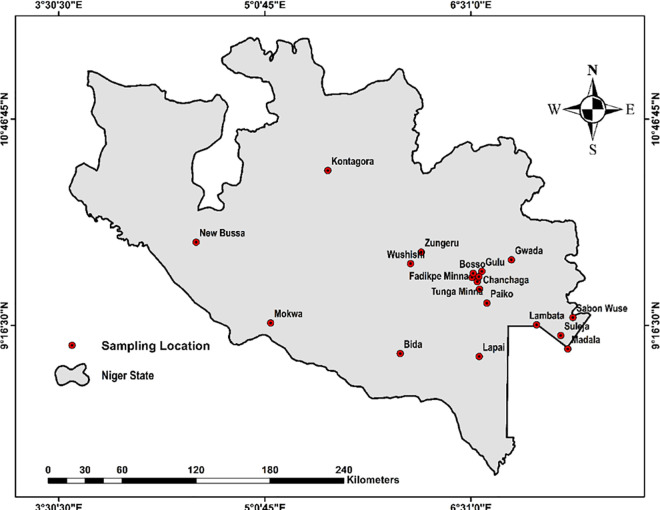
Sampling sites across Niger State, Nigeria.

The study population comprised individuals aged ≥ 18 years from diverse communities in Niger State (Fig 2) between August 2023 and August 2024. Participants were selected using a multistage random sampling method to ensure representative sampling across age groups, socioeconomic status, and residence type. Stool samples were obtained from consenting participants in a sterile 40 ml specimen container labeled with a unique ID following stringent aseptic procedures. Samples were immediately stored at 4°C in a portable cooler with ice packs and transported to the biosafety laboratory of the Trans-Saharan Disease Research Center (TDRC) at IBB University Lapai, Nigeria, within 4–6 h to ensure bacterial viability and prevent contamination. The collected samples were subsequently analyzed for the presence of *Salmonella* Typhi and multidrug resistance patterns. The inclusion of participants from various backgrounds facilitated the understanding of broader sociodemographic influences on typhoid fever prevalence.

### Sample size

The sample size for a large population was calculated using Cochran’s formula.


$$n_{0}=\frac{z^{2}x\;p\;x\;(1-p)}{e^{2}}\;$$

Where; e: desired level of precision, the margin of error; p: the fraction of the population (as percentage) that displays the attribute; z: the z-value, extracted from a z-table.

The sample size for the study was determined on the basis of the anticipated prevalence of typhoid fever in the region and the requirement for sufficient statistical power. In total, 624 participants were enrolled in this study. The age distribution of the participants was as follows: 18–27 years, 356 participants; 28–37 years, 204 participants; 38–47 years, 56 participants; and 48–77 years, 8 participants. This sample size provided an adequate representation of different age groups, ensuring the reliability and validity of the study findings.

### Isolation and identification of *Salmonella* Typhi

Stool samples were inoculated on *Salmonella Shigella* Agar (SSA) (Oxoid, UK) and Bismuth Sulfite Agar (BSA) (HiMedia, India) and then incubated at 37°C for 24 h. Colonies exhibiting *Salmonella* characteristics were stored short-term at +2°C and stored for long-term at −20°C to ensure viability for subsequent analysis. Presumptive *Salmonella* Typhi colonies on SSA appeared as colorless colonies with black centers, whereas those on BSA exhibited metallic black sheen with a dark periphery. Suspected isolates were further subjected to biochemical confirmation tests *S. Typhi*, including negative urease, lysine decarboxylase positivity, and lack of gas production from glucose. Positive isolates were sub-cultured on Nutrient Agar (NA) and incubated at 37°C for 24 h to obtain pure cultures, which were further transferred to Amies transport media and stored at −20°C for subsequent testing.

### Antibiotic sensitivity testing

Antimicrobial susceptibility testing was performed using the disk diffusion method in accordance with Clinical and Laboratory Standards Institute [14]. Isolates were tested for antibiotic sensitivity on Muller Hinton Agar (Oxoid, Basingstoke, UK) and Himedia Combi 78 plates. Sensitivity was tested against commonly used antibiotics, including ampicillin (10 μg), chloramphenicol (30 μg), ceftriaxone (30 µg), gentamicin (10 µg), cotrimoxazole (25 µg), levofloxacin (5 µg), netilmicin (30 µg), tetracycline (30 µg), amoxicillin/clavulanic acid (20/10 µg), and ofloxacin (5 µg) using standard antibiotic disks. When the initial tests indicated resistance, HiMedia G-I-Plus was used to confirm resistance patterns.

### Data collection and statistical analysis

Sociodemographic (age, sex, residence type, etc.) and clinical data (antibiotic usage and frequency of illness) were collected via structured questionnaires and interviews. Data was analyzed using multiple linear regression with percentage antibiotic sensitivity as the dependent variable to quantify the association between socio-clinical variables and antimicrobial response. Coefficients were interpreted as the average percent change in sensitivity associated with each predictor. Statistical significance was set at *P* < 0.05. IBM SPSS Statistics version 29 was used for the statistical analysis.

## Results

### Prevalence and sociodemographic associations

From the data collected, it was observed that out of the 624 participants, 228 (36.5%) tested positive for *Salmonella* Typhi, while 396 (63.5%) tested negative. Higher rates were observed among young adults (18–27 years) and residents in semi-urban areas. Gender analysis revealed a slight predominance in males. The sociodemographic data is summarized in Table 1. This study revealed significant associations between sociodemographic factors and typhoid prevalence. Young adults aged 18–27 years had a higher prevalence of typhoid fever. Residence type shaped disease prevalence, with semi-urban areas exhibiting the highest rate at 45.5%. Individuals using boreholes or tap water had significantly higher typhoid rates (25.6% and 57.1%, respectively). Higher infection rates were observed among secondary school graduates (37.8%).

**Table 1:** Community prevalence of *Salmonella* Typhi and demographic associations in Niger State.

Sociodemographic Factor	Category	Participants (n)	*S*. Typhi positive cases (n)	Prevalence (%)	p-Value
**Age**	18–27	356	190	83.3	0.0001
	28–37	204	25	11	
	38–47	56	12	5.2	
	48–77	8	1	0.4	
**Gender**	Male	328	120	52.6	0.587
	Female	296	108	47.4	
**Residence**	Urban	152	56	24.4	0.022
	Rural	188	69	30.1	
	Semi-urban	284	104	45.5	
**Water Sources**	Borehole	160	58.3	25.6	0.001
	Tap	356	130.1	57.1	
	Well	104	38.1	16.7	
	River	4	1.4	0.6	
**Toilet System**	Pit Latrine	241	88	38.4	0.0333
	Water Closet	326	120	52.5	
	Public Toilet	57	20	8.9	
**Occupation**	Student	376	137	60.0	0.005
	Civil Servant	168	61	26.8	
	Farmer	80	30	13.2	
**Education Level**	Primary	32	12	5.1	0.011
	Secondary	232	86	37.8	
	Technical/Vocational	184	58	25.6	
	Bachelors	104	38	16.7	
	Masters	60	29	12.8	
	None	12	5	1.9	

### Antibiotic sensitivity and resistance patterns

The antibiotic sensitivity patterns of the 228 isolates are presented in Table 2. The data indicated varying degrees of susceptibility to the 10 antibiotics tested, reflecting a spectrum of resistance within the isolates. None of the isolates (0%) was fully sensitive to any of the 10 antibiotics tested (100% sensitivity). One isolate (0.4%) exhibited complete resistance, showing no sensitivity to any of the antibiotics tested (0% sensitivity). The total number of isolates that were resistant to three or more antibiotics (multidrug-resistant) was 173 out of 228 isolates (76%). The antibiotic resistance patterns of the 228 isolates are presented in Table 3. The data revealed significant variations in the susceptibility of the isolates to the tested antibiotics. Netilmicin (NET) exhibited the highest sensitivity among the isolates. Amoxicillin/Clavulanic Acid (AMC) and ampicillin displayed complete resistance. All isolates (100%) were resistant, with no instances of sensitivity or intermediate response.

**Table 2:** Antibiotics sensitivity patterns of the 228 isolates.

Number of Antibiotics^*^	% Resistance^**^	No of isolates	Prevalence#
8	100	1	0.44%
7	87.5	5	2.19%
6	75	6	2.63%
5	62.5	9	3.95%
4	50	49	21.49%
3	37.5	103	45.18%
2	25	27	11.84%
1	12.5	28	12.28%
0	0	0	0.00%

* : Total number of antibiotics to which the 228 isolates exhibit sensitivity

** : The percentage of antibiotics (out of eight) to which the isolates are resistant

^#^: Percentage of isolates with a particular sensitivity pattern

**Table 3:** Antibiotic resistance patterns.

Antibiotic	Sensitivity (%)	Intermediate (%)	Resistant (%)	p-value
AMP (Ampicillin)	0	0	100	0.001
CHL (Chloramphenicol)	0	3.2	96.8	0.018
CTR (Ceftriaxone)	41.8	37.7	20.4	0.005
GEN (Gentamicin)	78.5	17.3	4.1	0.012
COT (Cotrimoxazole)	7.1	19.4	73.5	0.001
LE (Levofloxacin)	83.6	12.2	4.1	0.023
NET (Netilmicin)	86	12.2	2	0.018
TE (Tetracycline)	3.1	17.3	79.5	0.001
AMC (Amoxicillin/Clavulanic Acid)	0	0	100	0.001
OF (Ofloxacin)	82.6	13.2	4	0.001

### Association of clinical factors with multidrug resistance

The analysis revealed notable associations between multidrug resistance and clinical factors such as frequent self-prescription of antibiotics. Table 4 presents the association between various clinical factors and prevalence of multidrug resistance among the study participants. Among the participants who self-prescribed Amoxicillin/Clavulanic Acid, 32.1% exhibited multidrug resistance. The frequency of illnesses showed a strong association with multidrug resistance, with participants reporting monthly illnesses with the highest prevalence of multidrug resistance (28.7%). This was significantly higher than that in those who reported yearly illnesses (13.3%). The frequency of hospital visits also demonstrated a clear association with multidrug resistance. Participants who visited hospitals weekly had a higher prevalence of multidrug resistance (15.68%) than those who visited hospitals annually (3.99%). The frequency of hospitalization showed the strongest association with multidrug resistance, with participants hospitalized monthly having the highest prevalence of multidrug resistance (31.36%). This was significantly higher than in those hospitalized annually (15.71%). The frequency of illness and hospitalizations both showed a direct correlation with multidrug resistance, with higher frequencies associated with higher multidrug resistance prevalence. Notably, participants with a prior diagnosis of typhoid fever exhibited a higher prevalence of multidrug resistance (24.49%) than those with malaria (15.7%) or other illnesses (9.6%).

**Table 4:** Association of clinical factors with MDR.

Clinical Factor (n = 624)	Category	Participants (n)	Prevalence of MDR (%)
Self-prescribed Antibiotics	Ciprofloxacin	104	16.7
	Amoxicillin	60	9.6
	Amoxicillin/Clavulanate	200	32.1
Frequency of Illness	Monthly	112	28.7
	Quarterly	208	17.9
	Yearly	376	13.3
Frequency of Hospital Visits	Weekly	40	15.68
	Monthly	80	12.8
	Quarterly	128	7.83
	Yearly	304	3.99
Frequency of Hospitalizations	Monthly	40	31.36
	Quarterly	80	15.69
	Yearly	284	15.71
Previous Diagnoses	Malaria	336	15.7
	Typhoid	228	24.49
	Other Illnesses	60	9.6
Previous Antibiotic Use	Amoxicillin	80	12.8
	Ampicillin		
	Chloramphenicol		
	Gentamicin	76	12.2
	Tetracycline	312	50
	Ofloxacin	76	12.8
	Ciprofloxacin	80	12.2

### Multiple regression analysis of variables

Multiple regression analysis to assess the relationship between antibiotic sensitivity and the 14 predictor variables was summarized in Table 5. The coefficients (β), standard errors, and p-values for each independent variable are presented, indicating their influence on antibiotic sensitivity among the isolates. The frequency of hospitalization (β = −7.4016, p = 0.0278), recent antibiotic use (β = −0.0655, p = 0.0424), over-the-counter/self-prescribed antibiotics (β = −2.1665, p = 0.0582), and frequent use of antibiotics (β = 0.0277, p = 0.0598) were significant predictors of antibiotic sensitivity. Age (β = −0.4038, p = 0.258), sex (β = 1.2507, p = 0.7017), type of residence (β = 0.018, p = 0.9939), occupation (β = −2.2234, p = 0.4745), sources of drinking water (β = −1.7569, p = 0.3811), toilet system (β = −3.389, p = 0.2085), level of education completed (β = −0.8225, p = 0.526), frequency of falling sick (β = 0.8335, p = 0.5871), frequency of hospital visits (β = 1.8793, p = 0.2820), and frequency of previous antibiotic prescriptions (β = −0.2889, p = 0.2451) were not significant predictors.

**Table 5:** Multiple regression table-%Antibiotic sensitivity (dependent variable) vs other 14 variables (independent variables).

Predictor variables	Coefficients (β)	Standard Error	p-value
Age	−0.4038	0.3548	0.258
Gender	1.2507	3.2552	0.7017
Type Of Residence	0.018	2.3548	0.9939
Occupation	−2.2234	3.0954	0.4745
Sources Of Drinking Water	−1.7569	1.9956	0.3811
Toilet System	−3.389	2.6741	0.2085
Level Of Education Completed	−0.8225	1.2919	0.526
Freq. Of Falling Sick	0.8335	1.5289	0.5871
Freq. Of Hosp. Visit	1.8793	1.7357	0.2820
Freq. Of Hospitalization	−7.4016	3.3056	0.0278
Frequency Previous Antibiotic Prescribed	−0.2889	0.2468	0.2451
Over Counter/Self Prescribed Antibiotic	−2.1665	1.1281	0.0582
Frequent Use of Antibiotic	0.0277	0.2403	0.0598
Recently used Antibiotic	−0.0655	0.1986	0.0424

## Discussion

The observed prevalence of 36.5% (228 out of 624 participants) of *Salmonella* Typhi in communities in Niger State was notably high, suggesting a substantial burden of typhoid fever in the region. Similar findings have been reported from the southwestern region of Nigeria where invasive typhoidal *Salmonella* strains were identified in hospitalized patients [15]. This prevalence is also consistent with previous studies in sub-Saharan Africa, where poor sanitation, limited access to clean water, and inadequate healthcare infrastructure contributed to high typhoid incidence [1,5,16,17].

Sociodemographic factors may have contributed to typhoid prevalence. Here, we revealed significant associations between sociodemographic factors and typhoid prevalence. For instance, young adults aged 18–27 years showed a higher prevalence of typhoid fever, likely due to increased exposure to contaminated environments and riskier behaviors, elevating their exposure to pathogens. This trend agrees with findings in sub-Saharan Africa, where young populations often encounter infectious agents in crowded settings [18–20]. The male predominance, although not statistically significant, suggests that sex dynamics play a role in disease vulnerability. Previous studies suggest that behavioral factors likely play a secondary role in generating sex bias in infectious disease susceptibility [21], where males may exhibit higher rates due to societal roles, occupational exposure, and delayed healthcare-seeking behaviors [22].

Also, residence type shaped disease prevalence, with semi-urban areas exhibiting the highest rate at 45.5%. Han et al. [23] reported that type of residence plays a substantial role in diseases prevalence especially in older adults. This could reflect a transitional sanitation infrastructure that is insufficient to mitigate the transmission risk associated with urbanization. Previous investigations have similarly indicated a correlation between semi-urban environments and an elevated disease incidence [19]. Studies from within Nigeria, East Africa and South Asia highlight the need for effective Water, Sanitation, and Hygiene (WASH) initiatives to control disease transmission [18,24,25].

Furthermore, our research indicated that individuals using boreholes or tap water had significantly higher typhoid rates (25.6% and 57.1%, respectively). The association between specific water sources and typhoid prevalence emphasizes the importance of vectors for *Salmonella* Typhi transmission. These shared water sources may act as reservoirs for pathogens, correlating with studies that demonstrate links between contaminated drinking water and typhoid incidence [26–28]. Lower transmission rates are observed among individuals with protective behavior such as water cleaning practices. This is consistent with Sharma et al. [29] that proper water cleaning is important to reduce *Salmonella* transmission. This situation in Niger State highlights the need for improved water sanitation measures. Higher typhoid rates among water closet users compared to pit latrines and public toilet users could stem from contaminated water systems, a false sense of security, or shared facilities in densely populated areas. Intermittent water supply and improper storage practices can increase contamination risks, stressing the need for improved water management and hygiene education.

Similarly, occupational exposure, especially among students, is another crucial factor. The highest prevalence (60%) among students is possibly because of frequent interactions in school environments with inadequate sanitation. Joshua et al. [30] reported a 75.2% typhoid prevalence among students at Bingham University, Nigeria. Such elevated risk among students emphasizes the need for targeted intervention in educational institutions. Also, education level was also correlated with typhoid prevalence, as lower educational attainment often leads to limited health literacy and poorer sanitation practices. The higher infection rates among secondary school graduates (37.8%) suggest that increased exposure to communal settings may facilitate their spread. Studies have shown that individuals with higher education levels adopt better health practices, highlighting the protective role of health literacy in preventing typhoid transmission [31,32]. The lower infection rates in primary school leavers may be due to reduced exposure to high-risk environments and economic constraints. The observed patterns emphasize the necessity for comprehensive public health strategies aimed at enhancing the WASH infrastructure, improving sanitation facilities, and implementing targeted health education programs. Focusing on these areas, particularly in semi-urban and rural areas, can reduce typhoid fever prevalence and combat antibiotic resistance, thus contributing to long-term public health sustainability.

The prevalence of MDR strains is consistent with global trends, where antibiotic misuse has contributed to resistant bacteria emergence [9,10] and raises public health concerns. The antibiotic sensitivity and resistance patterns observed in this study highlight the evolving dynamics of *Salmonella* Typhi resistance in Niger State, Nigeria. The findings showed a prevalence of MDR strains, with 76% of isolates (173 out of 228) being resistant to three or more antibiotics. This level of resistance is concerning because none of the isolates displayed full sensitivity to antibiotics tested. Studies by Khadka et al. [33] and Xu et al. [34] detected similar high multidrug resistance *Salmonella* Typhi strains. One isolate (0.4%) exhibiting complete resistance to all tested antibiotics underscored the potential for untreatable infections. The absence of fully sensitive isolates emphasizes the need for regulation in antibiotic use and alternative treatment strategies. High resistance rates limit treatment options and increase the risk of severe disease outcomes [11].

The resistance patterns varied across the tested antibiotics. Netilmicin showed the highest sensitivity (98%), making it a potential candidate for empirical treatment. This aligns with studies from South Asia, where aminoglycosides have retained their efficacy against MDR strains [9]. Conversely, all isolates exhibited complete resistance (100%) to Amoxicillin/Clavulanic Acid, rendering it ineffective for treating typhoid fever in Niger State. This is consistent with the global trend of declining beta-lactam antibiotic efficacy [5]. High rates of resistance to first-line antibiotics, such as ampicillin (100%), chloramphenicol (96.8%), cotrimoxazole (73.5%) and tetracycline (79.5%), highlight their diminishing utility in managing typhoid fever [1]. Gentamicin (78.5%) and levofloxacin (83.6%) demonstrated relatively high sensitivities, indicating potential alternative treatment options for MDR infections. Ceftriaxone, a third-generation cephalosporin, showed moderate sensitivity (41.8%), with 20.4% of the isolates exhibiting full resistance. Ceftriaxone is often used as a second-line treatment for MDR typhoid fever [10]. The high resistance to cotrimoxazole (73.5%) and tetracycline (79.5%) further emphasizes the diminishing utility of these first-line antibiotics for typhoid treatment [1]. Whilst, levofloxacin and ofloxacin, both fluoroquinolones showed high sensitivity (83.6% and 82.6%, respectively), consistent with their previously reported effectiveness against MDR typhoid strains [12]. However, resistant isolates (4.1% for LE and 4% for OF) suggest emerging fluoroquinolone resistance, likely because of their widespread use in treatment [9].

These results agree with findings from other low- and middle-income countries (LMICs) where MDR *Salmonella* Typhi is increasingly prevalent. Studies in Pakistan, Kenya, and India have reported similar resistance patterns, particularly to Amoxicillin/Clavulanic Acid and Cotrimoxazole and emerging fluoroquinolone resistance [9,10,35]. However, the high sensitivity to netilmicin contrasts with regions where aminoglycosides show declining efficacy, indicating variations based on local antibiotic usage and healthcare practices [10]. The high prevalence of MDR isolates can be attributed to indiscriminate antibiotic use, poor sanitation, and inadequate public health infrastructure in Niger State [9,35,36].

Addressing these issues requires coordinated interventions to improve sanitation and hygiene, while optimizing antibiotic prescription practices. Increasing awareness of appropriate antibiotic use in healthcare settings and communities is essential to curb the development of resistance. Complete resistance to Amoxicillin/Clavulanic Acid and emerging resistance to fluoroquinolones necessitate re-evaluation of current treatment protocols. Public health campaigns should educate healthcare providers and the public about self-medication risks and the importance of adherence to prescribed antibiotic courses. Enhanced surveillance systems are critical for monitoring resistance patterns and for informing treatment guidelines.

The association between clinical factors and multidrug resistance in *Salmonella* Typhi isolates observed in this study provides critical insights into the drivers of antibiotic resistance in Niger State, Nigeria. The findings revealed the significant role of self-prescription of antibiotics, frequency of illness, and previous antibiotic use in shaping resistance patterns, underscoring the multifaceted nature of antimicrobial resistance in clinical settings. This is in line with Mohanty et al. [37] highlighting the role of self-prescription and rampant antibiotics misuse as important factors of antimicrobial resistance. These results highlight the urgent need for targeted interventions to address the misuse of antibiotics and improve regional healthcare practices.

Studies from similar contexts have shown that unrestricted access to antibiotics and self-medication practices are significant contributors to MDR patterns, as they accelerate selective pressure on pathogens to develop resistance [15,23]. Among the participants who self-prescribed Amoxicillin/Clavulanic Acid, 32.1% exhibited MDR, highlighting the implications of self-medication on treatment outcomes. This practice, often driven by a lack of access to healthcare or perceived efficiency of over-the-counter drugs, promotes an environment conducive to the development of resistance. The high prevalence of self-prescription agrees with findings from other LMICs, where limited access to healthcare and inadequate regulation of pharmacies facilitated the misuse of antibiotics [9].

Furthermore, the relationship between frequency of hospitalization, hospital visits, illness and repeated antibiotic exposure was investigated. Frequent hospitalizations are often indicative of severe or complicated infections, which are more likely to require broad-spectrum antibiotics and prolonged treatment, thereby creating ideal conditions for the development of resistance [7]. The high prevalence of MDR among frequently hospitalized individuals also suggests that healthcare settings may serve as reservoirs for resistant strains. The findings of our research are consistent with those of studies from other regions, where recurrent infections have been linked to the persistence of resistant strains due to incomplete or inappropriate treatment [12]. Interestingly, the prevalence of MDR associated with hospital visits was lower than that associated with illness frequency. This may reflect differences in healthcare practices, such as the use of more targeted or effective antibiotics in clinical settings compared with self-prescription. Garbern et al. [38] reported strong association between severe illness and high MDR frequency. Individuals who experience more frequent infections are likely to undergo multiple courses of antibiotic treatment, either through self-prescription or clinical treatment, which increases the risk of developing resistance [10]. Frequent illnesses may also indicate underlying vulnerabilities, such as compromised immunity or chronic exposure to contaminated environments, which further exacerbates the risk of MDR [5].

It is evident that the frequency of illness, hospital visits, and hospitalizations are interrelated but exhibit distinct patterns in their association with MDR. The magnitude of this association is greater for hospitalizations, likely due to more intensive antibiotic exposure in hospital settings. The findings of this study are consistent with those of other LMICs, where the frequency of illness and healthcare access has been identified as key drivers of antibiotic resistance [12]. For instance, a study in Pakistan found similar patterns of resistance among individuals with frequent hospitalizations and recurrent infections [9]. Similarly, studies in Kenya and India have documented the role of healthcare settings in the spread of MDR pathogens [5,7].

The multiple regression analysis in Table 5 provides insights into the factors influencing antibiotic sensitivity in *Salmonella* Typhi isolates from Niger State, Nigeria. The frequency of hospitalization showed a significant negative relationship with antibiotic sensitivity, indicating that patients with more frequent hospitalizations tended to have lower antibiotic sensitivity. This finding aligns with those of previous studies linking frequent hospitalizations to higher rates of antibiotic resistance [9,10]. This could be due to increased exposure to resistant pathogens during hospital stays or more severe chronic infections that are difficult to treat, highlighting the need for robust infection control measures in healthcare settings. Healthcare settings may also serve as reservoirs for resistant pathogens [5]. This underscores the need for enhanced infection control measures and responsible antibiotic use in hospitals.

The negative coefficient suggests that recent use of antibiotics, antibiotics misuse, age are associated with reduced sensitivity. Recent antibiotic use can disrupt the normal microbiota, creating an environment conducive to the proliferation of resistant strains [1]. This highlights how recent antibiotic exposure may lead to immediate changes in sensitivity patterns, supporting the idea of careful monitoring of antibiotic prescriptions to mitigate resistance. Also, self-prescription often leads to incomplete or inappropriate antibiotic use, creating ideal conditions for resistance development. This is consistent with studies from other LMICs, where self-medication and unregulated sale have been identified as key drivers of resistance [12]. Similarly, older individuals may have higher likelihood of previous antibiotic exposure, contributing to resistance [7].

The positive coefficient for gender and frequency of hospital visits suggest a trend toward increased antibiotic sensitivity among males and, possibly better healthcare access. This association emphasizes the importance of judicious antibiotic use and the need for healthcare providers to consider alternative treatment options when possible. Public health campaigns to educate the public about the dangers of unnecessary antibiotic use are also critical. Ultimately, stricter regulation of pharmacies and public health campaigns to educate about the dangers of self-medication are essential for addressing this issue.

### Public health implications

The findings of this study have significant implications for public health in Niger State, Nigeria, and similar regions experiencing high typhoid fever prevalence and multidrug resistance. The observed prevalence of 36.5% for *Salmonella* Typhi underscores the need for targeted interventions to reduce disease transmission and mitigate the spread of resistant strains. The high prevalence of MDR isolates (76%) emphasizes the need for responsible antibiotic use and improved infection control measures. High MDR cases in patients exacerbate pressure on an already strained health system, highlighting the urgency to address this issue. The association between water sources, toilet systems, and typhoid prevalence highlights the importance of improving WASH infrastructure. Public health campaigns should focus on educating communities about hygiene practices, risks of self-medication, and the importance of completing prescribed antibiotic courses. Healthcare providers should be trained in appropriate antibiotic use, and policies should restrict over-the-counter antibiotic sales.

## Conclusion

This study provides insights into sociodemographic and clinical factors influencing typhoid fever prevalence and multidrug resistance in Niger State, Nigeria. The high prevalence of *Salmonella* Typhi (36.5%) and substantial rate of MDR isolates (76%) emphasizes the need for targeted public health interventions. Associations were observed between water sources, sanitation practices, and typhoid transmission, as well as self-prescription and frequent hospitalizations in driving antibiotic resistance. The results emphasize improving WASH infrastructure, enhancing health education, and implementing antibiotic regulations to reduce disease transmission and mitigate resistance. By addressing root causes of typhoid fever and antibiotic resistance, Niger State can reduce disease burden, improve health outcomes, and contribute to global efforts to combat antimicrobial resistance. Future research should focus on longitudinal studies to monitor resistance patterns and evaluate intervention impacts, ensuring public health strategies remain effective and responsive to the evolving epidemiological landscape. Targeted interventions, such as antibiotic regulation, improved sanitation, and educational initiatives, are recommended to address the factors contributing to both disease prevalence and resistance.

### Limitations of the study

The study includes the application of a cross-sectional design which may limit causal inference; the consideration of antibiotic sensitivity modeling as a continuous outcome instead of a binary MDR classification which precludes the computation of odds ratios; and the use of self-reported clinical information which is prone to recall or reporting bias. In addition, although our analysis applied multivariable linear regression with several explanatory variables, there is no way to completely eliminate the possibility of residual confounding bias.

## Supporting information

S1 File Antibiotic sensitivity and resistance patterns. (XLSX)

S2 File A copy of study questionnaire and informed consent form. (PDF)
