# Supplementary material for: Sociodemographic and clinical factors influencing typhoid fever prevalence and multidrug resistance in Niger State, Nigeria
Source: PLoS One. 2025 Jul 8;20(7):e0327740. doi: 10.1371/journal.pone.0327740 (PMC12237014; doi:10.1371/journal.pone.0327740)
Supplement: S2 File — (PDF) [file pone.0327740.s002.pdf]

STUDY QUESTIONNAIRE/INFORMED CONSENT FORM

NRF TETFund Sponsored Research

Dear Sir/Ma,

We are conducting a research project on **"Molecular Epidemiology of *Salmonella enterica* Typhi and Their Antibiotic Resistance Pattern in North Central Nigeria"**. The study is aimed to determine the distribution pattern of molecular subtypes of the bacterium (*Salmonella* Typhi) that is the causative agent of Typhoid fever. It is also to determine the distribution of resistance genes for frequently used antibiotics (drugs) for the treatment of Typhoid fever in North-Central Nigeria. You are being asked to participate in this study because you satisfy the criteria set for inclusion into this study and we want to learn more about Typhoid fever and causes of its treatment difficulties and failures in our immediate community. We are asking you to participate in this study. If you agree to be part of the study, stool sample will be collected from you in the lab by a trained and certified medical lab scientist.

SECTION A: Socio demographic characteristics of respondents

1. Address/ location: ……………………………………………………….
2. Age: ……………………………
3. Gender: Male [ ] Female [ ]
4. Sate of residence: ……………………………………………… LGA: ………………………………………………………………………….
5. Type of place of residence: Rural [ ]; Semi-urban [ ]; Urban [ ]
6. Occupation: ……………………………………………………….
7. Sources of drinking water: ……………………………………………………………………………
8. Nature of Toilet system: ……………………………………………………………………………..
9. What is the highest degree or level of school you have completed?: Non-formal education [ ]; No schooling completed [ ]; Primary education [ ]; Secondary education [ ]; Technical/Vocational training [ ]; Bachelor's degree [ ]; Master's/Professional degree[ ]; Doctorate degree [ ]; Others……………………………………………..

SECTION B: Clinical characteristics of respondents

1. Frequency of falling sick: Weekly [ ]; Monthly [ ]; Quarterly [ ]; Yearly [ ]; N/A [ ]
2. Frequency of hospital visit: Weekly [ ]; Monthly [ ]; Quarterly [ ]; Yearly [ ]; N/A [ ]
3. Frequency of hospitalisation: Weekly [ ]; Monthly [ ]; Quarterly [ ]; Yearly [ ]; N/A [ ]
4. Frequent previous diagnoses: Malaria [ ]; Typhoid [ ]; Other diseases [ ];
5. Frequent previous antibiotics prescribed: ……………………….
6. Frequent over-the-counter/self-prescribed antibiotics used:……………………….
7. Frequent use of antibiotics: Daily [ ]; Weekly [ ]; Monthly [ ];Quarterly [ ]; Yearly[ ]; N/A [ ]
8. Recently used antibiotic: …………………………………………………………………………
9. List the different antibiotics previously used: …………………………………………………………………………………………………
   ………
10. Do you often follow through with prescriptions/dosage?: Yes [ ]; No [ ]
11. Blood/Stool culture for *Salmonella* Typhi: Positive [ ]; Negative [ ]

If you should have any questions about this study, please feel free to contact the Principal Investigator, Prof. Dickson Achimugu Musa on 08030557007

______________________________ ____________________ ______________

Participant's Name Signature Date

Consent obtained by:

______________________________ ____________________ ______________

Name Signature Date
